# Implementing efficient selective quantum process tomography of superconducting quantum gates on IBM quantum experience

**DOI:** 10.1038/s41598-022-07721-3

**Published:** 2022-03-07

**Authors:** Akshay Gaikwad, Krishna Shende, Arvind, Kavita Dorai

**Affiliations:** 1 Department of Physical Sciences, Indian Institute of Science Education and Research Mohali, Sector 81 SAS Nagar, Manauli, Punjab 14030 India; 2 Punjabi University, Patiala, Punjab 147002 India

**Keywords:** Information theory and computation, Quantum physics

## Abstract

The experimental implementation of selective quantum process tomography (SQPT) involves computing individual elements of the process matrix with the help of a special set of states called quantum 2-design states. However, the number of experimental settings required to prepare input states from quantum 2-design states to selectively and precisely compute a desired element of the process matrix is still high, and hence constructing the corresponding unitary operations in the lab is a daunting task. In order to reduce the experimental complexity, we mathematically reformulated the standard SQPT problem, which we term the modified SQPT (MSQPT) method. We designed the generalized quantum circuit to prepare the required set of input states and formulated an efficient measurement strategy aimed at minimizing the experimental cost of SQPT. We experimentally demonstrated the MSQPT protocol on the IBM QX2 cloud quantum processor and selectively characterized various two- and three-qubit quantum gates.

## Introduction

In the quest to build a real quantum computer, several difficulties need to be overcome, which include pure state initialization, implementing high fidelity quantum operations, performing efficient and noise-free measurements and protecting the quantum state against decoherence. Quantum state tomography (QST)^1^ and quantum process tomography (QPT)^2^ are standard tools that are extensively used for the characterization and benchmarking of quantum information processing devices and protocols.

Resource requirements for standard QST and QPT methods grow exponentially with increasing system size, and hence several novel methods have been designed that focus on simplifying and reducing experimental complexity such as maximum likelihood estimation^3^, adaptive quantum tomography^4^, self-guided tomography^5^, ancilla-assisted tomography^6^, compressed sensing tomography^7,8^, and least square optimization based tomography^9,10^. These novel tomography protocols have been experimentally demonstrated on various physical configurations such as NMR^11,12^, linear-optics^13^, NV-centers^14^, ion-trap based quantum processors^15^, photonic qubits^16^, and superconducting qubits^17–20^. It has been shown that sequential weak value measurement can be used to perform direct QPT of a qubit channel^21,22^. A unitary 2-design and a twirling QPT protocol have been used to certify a seven-qubit entangling gate on an NMR quantum processor^23^.

In recent years, researchers across the globe are engaged in building quantum systems of a larger register size termed noisy intermediate-scale quantum (NISQ) processors, such as the IBM quantum processor based on superconducting technology with 32 qubits, and NMR, ion-trap based quantum computers and linear optical photonic quantum processors which have achieved register sizes of 12, 10 and 14 qubits, respectively^24–26^. In some cases on such NISQ devices, instead of the complete characterization of the large-scale quantum process, one is only interested in a specific part, and the method used is termed selective quantum process tomography (SQPT)^27^ which allows us to perform partial process tomography. Specifically SQPT allows us to estimate single and selective elements of the process matrix, and provides the desired partial information of system dynamics. The first implementation of SQPT was reported using optics^28^, which involved the preparation of special quantum states called quantum 2-design states. Further developments in SQPT include the generalization of the SQPT protocol for arbitrary dimensions^29^ and an efficient protocol using an NMR quantum processor^30^ . However, the experimental complexity involved in performing SQPT is still high, and better strategies to implement SQPT need to be designed.

In this work we demonstrate a modified SQPT (MSQPT) protocol on a five-qubit IBM QX2 quantum information processor and use it to characterize several two- and three-qubit superconducting quantum gates. We propose a general quantum circuit for initial input state preparation to efficiently implement the MSQPT protocol. We implement an efficient measurement framework wherein detection is performed on only a single qubit. Our experimental results show that one can use the modified SQPT protocol to efficiently and selectively characterize the desired quantum process. We demonstrate that the MSQPT results can be further refined to construct the underlying true quantum process by solving a constrained convex optimization problem.

## Preliminaries

### Standard selective quantum process tomography

A quantum process denoted by the superoperator $\Lambda$ can be described using the Kraus operator representation^31^:

1 $$\begin{aligned} \Lambda (\rho )=\sum _{m,n} \chi _{mn} {E_m} \rho {E_n}^\dagger . \end{aligned}$$

with $\lbrace E_i \rbrace$ being a fixed set of basis operators, and $\rho$ being the quantum state evolving under $\Lambda$. The matrix $\chi$ with elements $\chi _{mn}$ characterizes the given quantum process $\Lambda$. Estimating the complete matrix $\chi$ is referred to as performing QPT of $\Lambda$. Full QPT is achieved by preparing a complete set of linearly independent quantum states $\lbrace \rho _i \rbrace$ and then letting them evolve under the quantum process under consideration^32^. However, sometimes it suffices to estimate specific elements of the $\chi$ matrix, a procedure referred to as selective QPT (SQPT), with an experimental complexity which is lower than the full QPT protocol^27^.

A specific element $\chi _{mn}$ of the process matrix can be determined by computing ‘average survival probabilities’ $F_{mn}$ as^28^:

2 $$\begin{aligned} F_{mn}=  {} \frac{1}{K} \sum _{j} \langle \phi _j \vert \Lambda (E_m^\dagger \vert \phi _j \rangle  \langle \phi _j \vert E_n)\vert \phi _j \rangle=  {} \frac{ D\chi _{mn}+\delta _{mn}}{D+1} \end{aligned}$$

where $\lbrace \vert \phi _i \rangle \rbrace$ are a set of quantum 2-design states^30^, *K* is their cardinality and *D* is the dimension of the Hilbert space.

From Eq. (2), it can be seen that in order to compute $F_{mn}$, one has to prepare the system in the state $(E_m^\dagger \vert \phi _j \rangle \langle \phi _j \vert E_n)$, let it pass through the given quantum process $\Lambda$ and then calculate the overlap with the original state $\vert \phi _j \rangle \langle \phi _j \vert$ for all quantum 2-design states. However, the operator $(E_m^\dagger \vert \phi _j \rangle \langle \phi _j \vert E_n)$ is in general not a valid density operator. Previous implementations^28^ have proposed an alternative way to resolve this issue, i.e. instead of the operator $(E_m^\dagger \vert \phi _j \rangle \langle \phi _j \vert E_n)$, the quantum system is prepared in the state $(E_m \pm E_n)^\dagger \vert \phi _j \rangle \langle \phi _j \vert (E_m \pm E_n)$ (which has to be divided by its trace for normalization), passed through the given quantum channel, the overlap with the original state is measured, the modified average fidelities $F_{mn}^{\pm }$ are computed and finally the real part of $F_{mn}$ is determined as:

3 $$\begin{aligned} \mathrm {Re}(F_{mn}) = \frac{F_{mn}^+-F_{mn}^-}{2} \end{aligned}$$

This approach is not experimentally viable as the number of experiments are quadrupled and to estimate a single element of the $\chi$ matrix we need to construct a large number of unitary operations corresponding to $(E_m + E_n)^\dagger \vert \phi _j \rangle \langle \phi _j \vert (E_m + E_n)$ and $(E_m - E_n)^\dagger \vert \phi _j \rangle \langle \phi _j \vert (E_m - E_n)$ for the real part and $(E_m + iE_n)^\dagger \vert \phi _j \rangle \langle \phi _j \vert (E_m + iE_n)$ and $(E_m - iE_n)^\dagger \vert \phi _j \rangle \langle \phi _j \vert (E_m - iE_n)$ for the imaginary part of the process matrix for all $\vert \phi _j \rangle$. For different values of *m* and *n*, we would again need to experimentally construct different sets of unitary operations which is a hard task to perform. Although the SQPT protocol is computationally less resource-intensive as compared to the standard QPT method, the number of experimental settings required to prepare the input states for computing a selected element of the process matrix is still quite high.

$$\begin{aligned} F_{mn}= & {} \frac{1}{K} \sum _{i,j,k} {^j\beta ^{mn}_{ki}} \mathbf{Tr} \left[ E_k \Lambda (E_i)\right] \\ \tilde{\rho _0}= & {} \frac{1}{D}E_0 \\ {\tilde{\rho }}_i= & {} \frac{1}{D}(E_i + I), \quad i>0 \\ {\bar{E}}_k^i= & {} \mathbf{Tr} [E_k \Lambda (E_i)] = D \mathbf{Tr} [E_k \Lambda (\tilde{\rho _i})] \\ {\bar{E}}_k^i= & {} \mathbf{Tr} [E_k \Lambda (E_i)] = D \mathbf{Tr} [E_k \Lambda (\tilde{\rho _i})] - {\bar{E}}_k^0, \quad i>0 \end{aligned}$$

### Protocol for modified selective quantum process tomography

$$\begin{aligned} \mathrm{Re}(F_{mn}) = \frac{F_{mn}^+-F_{mn}^-}{2} \end{aligned}$$

We propose a generalization of the SQPT method, namely the MSQPT protocol, which considerably reduces the experimental complexity of computing a desired element of the process matrix with high precision. We have designed a more efficient way of performing SQPT on an IBM quantum processor. We rewrite the operators $(E_m^\dagger \vert \phi _j \rangle \langle \phi _j \vert E_n)$ and $\Phi _j=\vert \phi _j \rangle \langle \phi _j \vert$ in Eq. (2) in terms of fixed basis operators $\lbrace E_i \rbrace$ as $E_m^\dagger \Phi _j E_n= \sum _{i} {^jc}_i^{mn}E_i$ and $\Phi _j=\sum _{k} {^je_k E_k}$ (with $^je_k\in {\mathbb {R}}$), which leads to the compact form:

4 $$\begin{aligned} F_{mn}= \frac{1}{K} \sum _{i,j,k} {^j\beta ^{mn}_{ki}} \mathbf{Tr} \left[ E_k \Lambda (E_i)\right] \end{aligned}$$

where the complex scalar quantities ${^j\beta ^{mn}_{ki}}={}^je_k \,{}^jc^{mn}_i$ can be computed analytically and do not depend upon the quantum process. It turns out that if we choose Pauli matrices as basis operators, then for given values of *m* and *n*, the tensor ${^j\beta ^{mn}_{ki}}$ is sufficiently sparse and most of its values are zero. We hence only need to compute $\mathbf{Tr} [E_k \Lambda (E_i)] \equiv {\bar{E}}_k^i$ for those values of *i* and *k* for which ${^j\beta ^{mn}_{ki}} \ne 0$. The sparsity of ${^j\beta ^{mn}_{ki}}$ is directly connected to the experimental complexity in terms of the number of coefficients ${\bar{E}}_k^i$ that need to be estimated.

The question now arises about the estimation of the coefficients ${\bar{E}}_k^i$. Given a set of operators $E_i$(*n*-qubit Pauli operators), one can associate a well defined (positive and unit trace) density operator $\tilde{\rho _i}$ with it as follows:

5 $$\begin{aligned} \tilde{\rho _0}= & {} \frac{1}{D}E_0\nonumber \\ {\tilde{\rho }}_i= & {} \frac{1}{D}(E_i + I), \quad i>0 \end{aligned}$$

It is easy to see that

6 $$\begin{aligned} {\bar{E}}_k^i = \mathbf{Tr} [E_k \Lambda (E_i)] = D \mathbf{Tr} [E_k \Lambda (\tilde{\rho _i})] \end{aligned}$$

Equation (6) hinges on the fact that the identity operator does not evolve under the process matrix $\Lambda$. This provides us with a way to experimentally estimate the desired coefficients ${\bar{E}}_k^i$, where we need to prepare the system in states ${\tilde{\rho }}_i$, let it evolve under the process $\Lambda$ and then measure $E_k$.

We note here that the identity operator is not preserved under the action of non-unital maps. In such cases, in order to perform modified SQPT of a given non-unital map, one needs to prepare the system in the state corresponding to the identity operator $E_0$ as well, which is the maximally mixed state denoted by $\tilde{\rho _{0}}$. Hence, for non-unital maps, Eq. (6) is modified as:

7 $$\begin{aligned} {\bar{E}}_k^i = \mathbf{Tr} [E_k \Lambda (E_i)] = D \mathbf{Tr} [E_k \Lambda (\tilde{\rho _i})] - {\bar{E}}_k^0, \quad i>0 \end{aligned}$$

where ${\bar{E}}_k^0 = D \mathbf{Tr} [E_k \Lambda (\tilde{\rho _0})]$, which can be experimentally computed by preparing the system in the ${\tilde{\rho }}_0$ state, passing it through the given non-unital quantum channel and then measuring the observables $E_k$. Once ${\bar{E}}_k^0$ is determined, the other desired coefficients ${\bar{E}}_k^i$ can be experimentally determined using Eq. (7). The computational efficiency of the MSQPT protocol is based on the fact that the total number of input states that are required to calculate the average survival probabilities (Eq. 2) is much fewer as compared to the SQPT method, as a single unitary operator is applied simultaneously on all system qubits to prepare the input state. Furthermore, only a single detection is required at a time, which reduces the number of readouts required to determine a specific element of the process matrix, further reducing the experimental complexity of the protocol.

**Figure 1:**
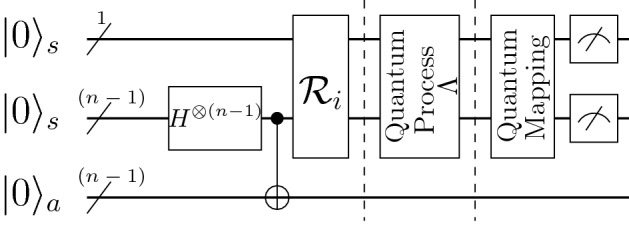
The quantum circuit to acquire data to perform an *n*-qubit MSQPT. The symbol ‘/’ through the input wire represents a multiqubit quantum register. The first and the second quantum registers collectively represent the system qubits (denoted by $\vert 0 \rangle _s$), and the third quantum register represents the ancilla qubits (denoted by $\vert 0 \rangle _a$). The first block prepares the desired pure input state $\vert \Psi _i\rangle$. The unitary gate ${\mathscr {R}}_i$ is then applied on the system qubits. The second block represents the unknown quantum process which is to be applied to the system qubits and the last block represents the measurement settings to compute the expectation value of the desired observable.

The quantum circuit to implement the *n*-qubit MSQPT protocol is given in Fig. 1. The symbol ‘/’ through the input wire represents a multiqubit quantum register. The first quantum register contains a single qubit while the second and third quantum registers comprise $n-1$ qubits, respectively. The first and the second quantum registers collectively represent the system qubits denoted by $\vert 0 \rangle _s$, while the third quantum register represents the ancilla qubits denoted by $\vert 0 \rangle _a$. The first block prepares the desired pure input state $\vert \Psi _i\rangle$, where $H^{\otimes (n-1)}$ is applied on the second register followed by $n-1$ CNOT gates, with the control being at the second quantum register and the target being at the third quantum register. The unitary gate ${\mathscr {R}}_i$ is then applied on the system qubits, where the columns of the unitary operation ${\mathscr {R}}_i$ are the normalized eigenvectors of the density matrix ${\tilde{\rho }}_i$. For non-unital quantum channels, in order to prepare the *n*-qubit system in the maximally mixed state $\tilde{\rho _{0}}$,one needs an extra ancillary qubit as compared to preparing other $\tilde{\rho _{i}}$ $(i>0)$. One has to prepare the joint system (main system qubits+ancilla qubits) in the state: $\vert \Psi _0 \rangle = \sum _i (\vert e_{i} \rangle \otimes \vert e^{\prime }_{i} \rangle ) /\sqrt{2^n}$, where $\vert e_{i} \rangle$ and $\vert e^{\prime }_{i} \rangle$ are computational basis vectors of the system qubits and the ancilla qubits, respectively. It is to be noted that $\vert \Psi _0 \rangle$ has a Schmidt rank greater than 1, which demonstrates that the state is entangled. For example, for a two-qubit system, the state of the combined system (system + ancilla) is:

8 $$\begin{aligned} \vert \Psi _0 \rangle =\frac{\vert 00 \rangle \vert 00 \rangle + \vert 01 \rangle \vert 01 \rangle + \vert 10 \rangle \vert 10 \rangle + \vert 11 \rangle \vert 11 \rangle }{2} \end{aligned}$$

For an *n*-qubit system, the state $\tilde{\rho _{0}}$ corresponding to the identity operator can be prepared by applying *n* Hadamard gates on *n* system qubits and then applying *n* CNOT gates with the system qubits being the control and ancilla qubits being the target. The unitary gate ${\mathscr {R}}_0$ is an *n*-qubit identity operation. The rest of the protocol and the quantum circuit given in Fig. 1 remains unaltered. The second block represents the unknown quantum process which is to be characterized and the last block represents the measurement settings to compute the expectation values of the desired observables. Note that in the third block, after the appropriate quantum mapping, only a single detection is performed at a time, to measure a desired observable.

In order to represent a valid quantum map, the $\chi$ matrix should satisfy following conditions^33^: (1) $\chi = \chi ^{\dagger }$, (2) $\chi \ge 0$ and (3) $\sum _{m,n}\chi _{mn}E_m^{\dagger }E_n = I$. Using the MSQPT method, the $\chi$ matrix is Hermitian by construction, however there is no guarantee that it will satisfy the last two conditions. One can use the constrained convex optimization (CCO) technique^10^ to obtain a valid $\chi _{\mathrm{cco}}$ matrix from $\chi _{\mathrm{msqpt}}$ as follows:

9a $$\begin{aligned}&\!\min _{{{\chi _{\mathrm{cco}}}}}&\qquad&\Vert \chi _\mathrm{msqpt}-\chi _{\mathrm{cco}}\Vert _{l_2} \end{aligned}$$

9b $$\begin{aligned}&\text {subject to}&\chi _{\mathrm{cco}} \ge 0, \end{aligned}$$

9c $$\begin{aligned}&&\sum _{m,n}\chi _{mn}^\mathrm{cco}E_m^{\dagger }E_n = I. \end{aligned}$$

where $\chi _{\mathrm{msqpt}}$ is the experimentally obtained process matrix using the MSQPT protocol and $\chi _{\mathrm{cco}}$ is the variable process matrix which represents the underlying true quantum process.

### State preparation and unitary operator construction

We note here that for an *n*-qubit system, all density operators $\tilde{\rho _i}$ in Eq. (5) represent mixed states (except for $n=1$). We hence require ancillary qubits to experimentally prepare the quantum system in the state $\tilde{\rho _i}$.

It turns out that for an *n*-qubit system, all non-zero eigenvalues of the operator $\tilde{\rho _i}$ in Eq. (5) are the same and are equal to $1/2^{n-1}$. Let $\lbrace \vert u_1^i \rangle , \vert u_2^i \rangle , \vert u_3^i \rangle ,\ldots , \vert u_{2^{n-1}}^i \rangle \rbrace$ represent the complete set of normalized eigenvectors of the operator $\tilde{\rho _i}$ corresponding to its non-zero eigenvalues. The state of the combined system (system $+$ ancilla) we need to prepare is given by:

10 $$\begin{aligned} \vert \Psi _i \rangle= & {} \frac{ \vert u_1^i \rangle \vert a_1\rangle + \vert u_2^i \rangle \vert a_2\rangle +\cdots + \vert u_{2^{n-1}}^i \rangle \vert a_{2^{n-1}}\rangle }{\sqrt{2^{n-1}}} \end{aligned}$$

where $\vert a_i\rangle$ are the basis states of the ancilla qubits. Note that in general $\vert \Psi _i\rangle$ represents an entangled state. After tracing over the ancillary qubits, the system will be in the desired state $\tilde{\rho _i}$.

The unitary operator $U^i$, such that $U^i \vert 0 \rangle ^{sys} \vert 0 \rangle ^{ancilla} = \vert \Psi _i \rangle$ can be constructed as follows:

1. Apply a Hadamard gate on ($n-1$) system qubits; $2^{n-1}$ number of states will be in a superposition state while the ancilla qubits will be in the state $\vert 0 \rangle ^{ancilla}$.
2. Apply CNOT gates with the system qubits being the controls and ancilla qubits being the target. We hence have $\vert 0 \rangle ^{ancilla} \longrightarrow \vert a_1^i \rangle$, $\vert 0 \rangle ^{ancilla} \longrightarrow \vert a_2^i \rangle$, and so on.
3. Map the computational basis states of the system qubits to the eigenvectors of $\tilde{\rho _i}$ using the unitary gate ${\mathscr {R}}_i$, where the columns of ${\mathscr {R}}_i$ are the normalized eigenvectors of $\tilde{\rho _i}$ Eq. (5). Note that the column position of eigenvectors depends on which computational basis vector we want to map onto which eigenvector. The combined system (system $+$ ancilla qubits) will be in the $\vert \Psi _i\rangle$ state.
4. Repeat the steps [1–3] to prepare other states $\tilde{\rho _i}$.

**Figure 2:**
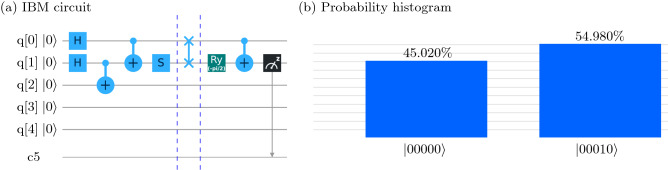
(**a**) The IBM quantum circuit to perform MSQPT of a two-qubit SWAP gate. The first block prepares the three-qubit input state $\vert \psi _{6} \rangle$. The quantum process corresponding to the two-qubit SWAP gate is applied in the second block and in the last block, the quantum map $U_{13}=$CNOT$_{12}$. $R_y(-\frac{\pi }{2})$ is applied to compute $\mathrm{Tr}(\sigma _z \otimes \sigma _x \Lambda (\tilde{\rho _6}))$ by detecting the second qubit in the $\sigma _z$ basis. (**b**) The histogram representing statistical results after implementing the quantum circuit given in (**a**), 4096 times. The values $p_0 = 0.4502$ and $p_1 = 0.5498$ represent the probabilities of obtaining the second qubit in the $\vert 0 \rangle$ and the $\vert 1 \rangle$ state, respectively.

## Results and discussion

The IBM quantum processor is based on superconducting qubits and is freely available through the cloud^34–36^, and has been used to demonstrate various quantum protocols^37,38^. More details about the architecture of the IBM QX2 processor and the topology of superconducting qubits are given in^39^ and information about the form of the Hamiltonian and important relaxation parameters can be found in^40,41^. We use the five-qubit IBM QX2 processor to demonstrate the MSQPT protocol described in the previous section. The system is prepared in an input state corresponding to all qubits being in the $\vert 0 \rangle$ state. After the gate implementation, projective measurements are performed in the Pauli $\sigma _z$ basis and the quantum circuit is implemented multiple times to compute the Born probabilities. The IBM quantum architecture requires a pure quantum state as an input state and only allows the implementation of unitary operations. We hence utilize ancillary qubits to prepare the system in a mixed state and to simulate non-unitary evolution.

We implement the MSQPT protocol corresponding to two- and three-qubit gates and element wise construct the corresponding full $\chi$ matrices. In all the cases considered, we use the experimentally constructed $\chi _{\mathrm{msqpt}}$, solve the CCO problem (Eq. 9a) and obtain $\chi _{\mathrm{cco}}$, which represents the underlying true quantum process. The fidelity of experimentally implemented quantum gates is computed using the measure^14^:

11 $$\begin{aligned} {{\mathscr {F}}}(\chi ^{}_{\mathrm{exp}},\chi ^{}_{\mathrm{the}})= & {} \frac{|\mathrm{Tr}[\chi ^{}_{\mathrm{exp}}\chi _{\mathrm{the}}^\dagger ]|}{\sqrt{\mathrm{Tr}[\chi _{\mathrm{exp}}^\dagger \chi ^{}_{\mathrm{exp}}] \mathrm{Tr}[\chi _{\mathrm{the}}^\dagger \chi ^{}_{\mathrm{the}}]}} \end{aligned}$$

To validate our circuits, we also theoretically simulate the MSQPT protocol on the IBM processor and obtain $\chi _{\mathrm{sim}}$. The fidelity of the simulated quantum gates is computed by using a similar measure as given in Eq. (11).

### MSQPT of two-qubit quantum gates

For two qubits, we need to prepare 15 input (mixed) states ${\tilde{\rho }}_i$ (Eq. 5) corresponding to all the Pauli operators $E_i$. For all ${\tilde{\rho }}_i$s, it turns out that out of four eigenvalues, only two eigenvalues are non-zero ($\lambda _1 = \lambda _2 = 1/2$). Let $\vert v^i_1 \rangle$ and $\vert v^i_2 \rangle$ represent the normalized eigenvectors of the operator $\tilde{\rho _i}$ corresponding to $\lambda _1$ and $\lambda _2$, respectively. To perform MSQPT of two qubits on the IBM computer, we use one ancillary qubit and prepare three-qubit input (pure) states of the form:

12 $$\begin{aligned} \vert \psi _i \rangle = \frac{\vert v^i_1 \rangle \vert 0 \rangle + \vert v^i_2 \rangle \vert 1 \rangle }{\sqrt{2}} \end{aligned}$$

All 15 three-qubit pure input states $\vert \psi _i \rangle$ corresponding to $E_i$ are listed below:

$$\begin{aligned}{}&\vert \psi _1 \rangle =[(0,1,0,1,1,0,1,0)/2]^{T}, \\&\vert \psi _2 \rangle =[(0,-i,0,1,-i,0,1,0)/2]^T, \\&\vert \psi _3 \rangle =[(1,1,0,0,0,0,0,0)/\sqrt{2}]^T, \\&\vert \psi _4 \rangle =[(0,-1,1,0,0,-1,1,0)/2]^T, \\ {}&\vert \psi _5 \rangle =[(1,0,0,1,0,1,1,0)/2]^T, \\ {}&\vert \psi _6 \rangle =[(-i,0,0,1,0,-i,1,0)/2]^T, \\ {}&\vert \psi _7 \rangle =[(0,1,-1,0,0,1,1,0)/2]^T, \\ {}&\vert \psi _8 \rangle =[(0,-1,-i,0,0,-i,1,0)/2]^T, \\ {}&\vert \psi _9 \rangle =[(-i,0,0,1,0,i,1,0)/2]^T, \\ {}&\vert \psi _{10} \rangle =[(-1,0,0,1,0,1,1,0)/2]^T, \\ {}&\vert \psi _{11} \rangle =[(0,1,1,0,0,1,1,0)/2]^T, \\ {}&\vert \psi _{12} \rangle =[(1,0,0,1,0,0,0,0)/\sqrt{2}]^T, \\ {}&\vert \psi _{13} \rangle =[(0,1,0,1,-1,0,1,0)/2]^T, \\ {}&\vert \psi _{14} \rangle =[(0,-i,0,1,i,0,1,0)/2]^T, \\ {}&\vert \psi _{15} \rangle =[(1,0,0,0,0,0,0,1)/\sqrt{2}]^T \end{aligned}$$

As an illustration, the IBM quantum circuit for implementing MSQPT of a two-qubit SWAP gate, corresponding to the quantum state $$\vert \psi _6 \rangle $$ and the observable $$E_{13} = \sigma _z \otimes \sigma _x$$, is given in Fig. 2. The system qubits are denoted by *q*[0] and *q*[1] (the first and second qubit, respectively) while the ancilla qubit is denoted by *q*[2]. To prepare the system in the pure state $$\vert \psi _6 \rangle $$, the unitary operation $$U^6 = S_2.$$ CNOT$$_{12}. $$CNOT$$_{23}.$$ H$$_2.$$ H$$_1$$ is applied on the initial state $$\vert 000 \rangle $$ in the first block. In the second block, the quantum process $$(\Lambda _{system} \otimes I_{ancilla})$$ corresponding to a two-qubit SWAP gate is implemented on the system qubits. In the last block, the quantum map corresponding to the unitary operation $$U_{13}=$$ CNOT$$_{12}.R_y(-\frac{\pi }{2})$$ is used to transform the output state and determine $$E_{13}=\langle \sigma _z \otimes \sigma _x \rangle $$ by measuring the second qubit in the $$ \sigma _{z} $$ basis^30^. The quantity corresponding to $$\mathrm{Tr}(\sigma _z \otimes \sigma _x \Lambda (\tilde{\rho _6}))$$ is experimentally computed, which is equivalent to $$\mathrm{Tr}(\sigma _{2z} U_{13}(\Lambda (\tilde{\rho _6})){U_{13}}^{\dagger })$$. Using Eq. (5) we obtain:

13 $$\begin{aligned} \mathrm{Tr}(\sigma _z \otimes \sigma _x \Lambda (\sigma _x \otimes \sigma _y ))= &\, {} 4 \mathrm{Tr}(\sigma _z \otimes \sigma _x \Lambda (\tilde{\rho _6})) \nonumber \\= &\, {} 4 \mathrm{Tr}(\sigma _{2z} U_{13}(\Lambda (\tilde{\rho _6})){U_{13}}^{\dagger }) \nonumber \\ \end{aligned}$$

One can thus efficiently compute all the $\langle E_k^i \rangle$ (Eq. 4) and estimate the corresponding average survival probabilities $F_{mn}$. The list of all unitary operations $U_i$ corresponding to all quantum maps which transform output states in order to determine $\langle E_k \rangle$ by detecting either of the system qubits in the $\sigma _z$ basis (*i.e.* by measuring either $\langle \sigma _{1z} \rangle$ or $\langle \sigma _{2z} \rangle$) is given in^30^.

**Figure 3:**
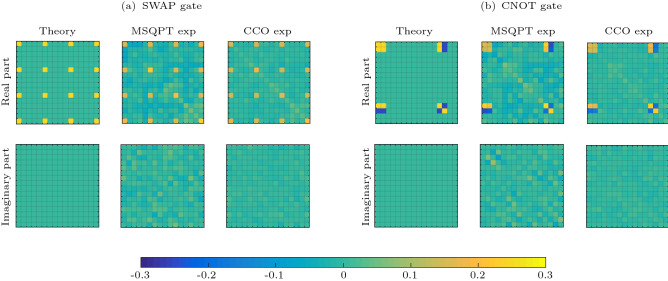
Matrix plots corresponding to the real and imaginary parts of the (**a**) $\chi$ matrix for the SWAP gate and (**b**) $\chi$ matrix for the CNOT gate. The first column represents the theoretically constructed process matrix $\chi _{\mathrm{the}}$, while the second and third columns represent $\chi _{\mathrm{msqpt}}$, and $\chi _{\mathrm{cco}}$, respectively. The top row represents the real part of the process matrix while the bottom row represents the imaginary part of the process matrix. The matrix plots were generated using MATLAB^42^.

**Table 1:** Experimental complexity and the number of ancillary qubits required for the implementation of two-qubit MSQPT, SQPT and standard QPT protocols.

	MSQPT	SQPT	Standard QPT
Preparations	15	80	15
Readouts	60	240	225
Ancilla qubits	1	0	0

The $16\times 16$ grid matrix plots in Fig. 3a represent $\chi$ matrix corresponding to the two-qubit SWAP gate, where the position of the specific grid represents the corresponding element of the $\chi$ matrix, while its color represents its value. For instance, the first yellow square in the matrix plot in Fig. 3a denotes the element $\chi _{11}=0.25$ of the theoretically constructed process matrix $\chi _{\mathrm{the}}$. Only 16 yellow squares have non-zero values in the theoretically constructed matrix plot for the SWAP gate. The second and third columns represent matrix plots corresponding $\chi _{\mathrm{msqpt}}$, and $\chi _{\mathrm{cco}}$ respectively obtained by implementing MSQPT protocol on IBM QX2 processor. The differences in the theoretically computed and experimentally obtained matrix plots reflect errors due to decoherence and statistical and systematic errors while preparing the initial input state. The color grids in the matrix plots in Fig. 3 in the third column (CCO experimental) have a smaller deviation as compared to the matrix plots in the second column (MSQPT experimental). This improved fidelity implies that one can use the MSQPT data to solve CCO problem and reconstruct the full process matrix more accurately. The experimental fidelity of $\chi _{\mathrm{msqpt}}$ for the SWAP gate (Fig. 3a) turned out to be 0.799, while the improved fidelity of $\chi _{\mathrm{cco}}$ turned out to be 0.929. We also computed the process matrices for the two-qubit CNOT gate and the corresponding matrix plots are shown in Fig. 3b. The experimental fidelity of $\chi _{\mathrm{msqpt}}$ for the CNOT gate turned out to be 0.828, while the improved fidelity of $\chi _{\mathrm{cco}}$ turned out to be 0.953. We obtained fidelities of ${\mathscr {F}}(\chi _{\mathrm{sim}})\ge 0.99$ for all the quantum gates, which ensures that all the quantum circuits are correct. The fidelity values of ${\mathscr {F}}(\chi _{\mathrm{cco}})\ge 0.9$ shows that one can retrieve the full dynamics of the quantum process with considerably high precision by solving the optimization problem (Eq. 9a) using the experimentally constructed full $\chi _{\mathrm{msqpt}}$ matrix.

The standard QPT protocol is based on the linear inversion method and requires the preparation of 15 linearly independent input states and further requires the state tomography of each output state. Hence the total number of readouts to determine a specific element of the two-qubit process matrix with high precision, using the standard QPT protocol, is $15 \times 15 = 225$. The SQPT protocol uses quantum two-design states as initial input states and further requires a quantum operation to prepare the system in the desired state. Determining the real and imaginary parts of $F_{mn}$ respectively requires a total of 80 state preparations. Further, to estimate the overlap with original state $\vert \phi _j \rangle \langle \phi _j \vert$ (Eq. 2), three readouts need to be performed (as there are three non-zero coefficients in the decomposition of $\Phi _j$). Hence the total number of readouts to determine a specific element of the two-qubit process matrix with high precision, using the SQPT protocol, is $80 \times 3 =240$. In the MSQPT protocol, the average survival probabilities can be computed quite efficiently as the total number of states that need to be prepared are only 15, there are only 12 readouts per mutually unbiased basis (MUB) set, and there are 5 MUB sets which form a complete set of quantum 2-design states. Hence the total number of readouts to determine a specific element of the two-qubit process matrix with high precision, using the MSQPT protocol, is only $12 \times 5 = 60$. The experimental complexity and number of ancilla qubits required to determine a specific element of the process matrix with high precision for a two-qubit system, using the MSQPT method, are compared with the standard QPT and SQPT methods in Table 1.

**Figure 4:**
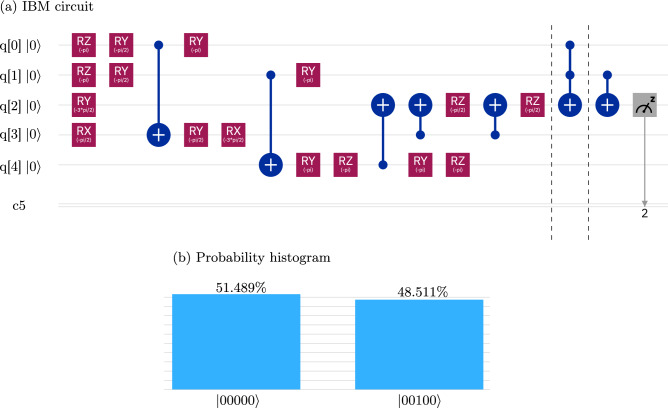
(**a**) The IBM quantum circuit to perform MSQPT of a three-qubit Toffoli gate. The first block prepares the five-qubit input state $$\vert \Omega _{50} \rangle $$. The quantum process corresponding to the Toffoli gate is applied in the second block, and in the last block, the quantum map $$U_{15}= $$CNOT$$_{23}$$ is applied to compute $$\mathrm{Tr}(I \otimes \sigma _z \otimes \sigma _y \Lambda ({\tilde{\rho }}_{50}))$$ by detecting the third qubit in the $$\sigma _z$$ basis. (**b**) The histogram represents statistical results after implementing the quantum circuit given in (**a**), 4096 times. The values $$p_0 = 0.51489$$ and $$p_1 = 0.48511$$ represent the probabilities of obtaining the third qubit in the $$\vert 0 \rangle $$ and the $$\vert 1 \rangle $$ state, respectively.

### MSQPT of three-qubit quantum gates

To perform MSQPT on a three-qubit system, we need to prepare 63 input (mixed) states $\tilde{\rho _i}$ corresponding to all the three-qubit Pauli operators $E_i$. It turns out that for all $\tilde{\rho _i}$, out of 8 eigenvalues only 4 are non zero and are equal to 1/4. Let $\vert u_1^i \rangle$, $\vert u_2^i \rangle$, $\vert u_3^i \rangle$ and $\vert u_4^i \rangle$ be the 4 eigenvectors of $\tilde{\rho _i}$ with non-zero eigenvalues. In order to prepare the system in the any of the $\tilde{\rho _i}$ states, we first need to prepare a five-qubit pure state:

14 $$\begin{aligned}&\vert \Omega _i \rangle = \frac{\vert u^i_1 \rangle \vert 00 \rangle + \vert u^i_2 \rangle \vert 01 \rangle + \vert u^i_3 \rangle \vert 10 \rangle + \vert u^i_4 \rangle \vert 11 \rangle }{2} \end{aligned}$$

After tracing out the two ancilla qubits, the three system qubits are in the state $\tilde{\rho _i}$, i.e., $\mathrm{Tr}^{ancilla}(\vert \Omega _i \rangle \langle \Omega _i \vert ) = \tilde{\rho _i}$. The list of all five-qubit pure input states $\lbrace \vert \Omega _i \rangle \rbrace$ is given in Supplementary Information.

**Figure 5:**
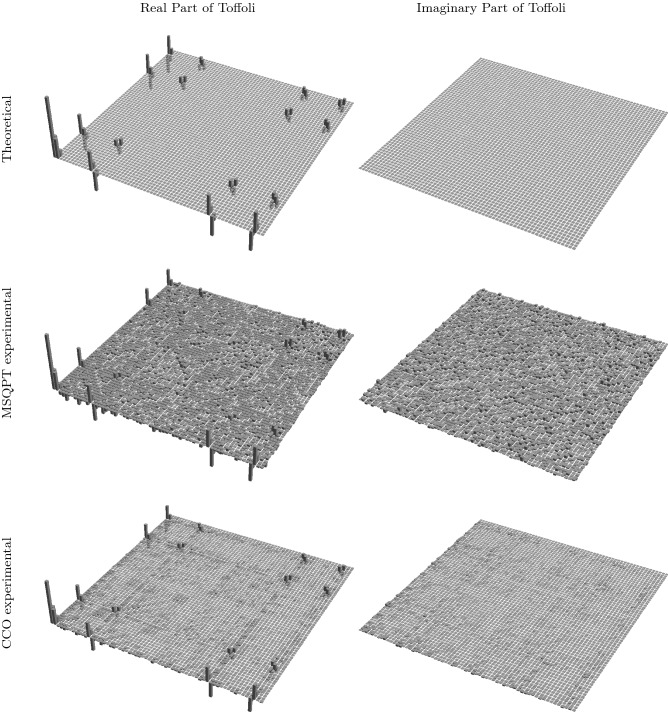
Tomographs corresponding to the three-qubit Toffoli gate, with the first and second columns representing the real and imaginary part of the $\chi$ matrix, respectively. The first row represents the theoretically constructed $\chi$ matrix while the second and third rows represent the experimentally constructed $\chi$ matrix obtained by implementing the MSQPT and the CCO protocols, respectively. The tomographs were generated using Mathematica^43^.

**Table 2:** Experimental complexity and the number of ancilla qubits required for the implementation of three-qubit MSQPT, SQPT and standard QPT protocols.

	MSQPT	SQPT	Standard QPT
Preparations	63	288	63
Readouts	504	2016	3969
Ancilla qubits	2	0	0

Preparation of the five-qubit input state requires finding the correct decomposition of the unitary operator $${\mathscr {R}}_i$$ (Fig. 1) in terms of CNOT gates and single-qubit rotations. We note here that finding the decomposition of a general unitary operation $${\mathscr {R}}_i$$ is not an easy task. The complexity of the implementation of a given unitary operation primarily depends on the limitations of the quantum hardware being used. The IBM processor that we have used allows only a limited number of quantum gates to be implemented directly. Thus the implementation of a general unitary operation on the IBM processor involves its efficient decomposition as a sequence of the available quantum gate and then its implementation. Particularly in the context of MSQPT, the construction of unitary operators $${\mathscr {R}}_i$$ is system-specific and finding a general algorithm to experimentally implement $${\mathscr {R}}_i$$ on a given physical system is a research direction that requires more efforts. There are several techniques available to decompose a given unitary operation into a universal set of quantum gates^44–47^. In this study we have used the Mathematica package *UniversalQCompiler*^46,47^ as an optimization tool to prepare the input state $$\vert \Omega _i \rangle $$ from the initial state $$\vert 00000 \rangle $$. The quantum circuit to perform MSQPT of a three-qubit Toffoli gate is given in Fig. 4, corresponding to the five-qubit pure input state $$\vert \Omega _{50} \rangle $$ and the observable $$E_{15} = I \otimes \sigma _{z} \otimes \sigma _{y}$$. The system qubits are denoted by *q*[0], *q*[1] and *q*[2], while the ancilla qubits are denoted by *q*[3] and *q*[4], respectively. The first block in Fig. 4 prepares the five-qubit pure input state $$\vert \Omega _{50} \rangle $$ while the second block represents the action of the Toffoli gate on the system qubits and the last block represents the action of the quantum map corresponding to the unitary operation $$U_{15}= $$CNOT$$_{23}$$. A measurement is made on the third qubit in the $$\sigma _{z}$$ basis, to compute the quantity $$\mathrm{Tr}(\sigma _{3z} U_{15}(\Lambda ({\tilde{\rho }}_{50})){U_{15}}^{\dagger })$$, and obtain:

15 $$\begin{aligned} \mathrm{Tr}(E_{15} \Lambda (E_{50} ))= & {} \, 8 \mathrm{Tr}(E_{15} \Lambda ({\tilde{\rho }}_{50})) \nonumber \\= & {} \,8\mathrm{Tr}(\sigma _{3z} U_{15}(\Lambda ({\tilde{\rho }}_{50})){U_{15}}^{\dagger }) \end{aligned}$$

All the $\mathrm{Tr}(E_k \Lambda (E_i))=\langle E^i_k\rangle$ can be computed in a similar fashion, corresponding to the desired average survival probability $F_{mn}$. The list of all unitary operations $U_{i}$, corresponding to all quantum maps for the three-qubit system can be found in^48^. The experimentally obtained $64\times 64$ dimensional $\chi$ matrix corresponding to the three-qubit Toffoli gate is depicted in Fig. 5 as a bar plot, where the first and second columns represent the real and imaginary parts of the $\chi$ matrix, respectively. The first row denotes the theoretically constructed process matrix $\chi _{\mathrm{the}}$, while the second and third rows represent the experimentally constructed process matrices $\chi _{\mathrm{msqpt}}$ and $\chi _{\mathrm{cco}}$, respectively. The experimental gate fidelity for $\chi _{\mathrm{msqpt}}$ turns out to be 0.589, while the much improved experimental gate fidelity obtained for the case of $\chi _{\mathrm{cco}}$ turns out be 0.946. To ensure the correctness of the circuits, we also simulated all the circuits on the IBM simulator, with a simulation fidelity of 0.98.

The total number of readouts to determine a specific element of the three-qubit process matrix with high precision, using the standard QPT protocol, is $63 \times 63 = 3969$. For three qubits, the cardinality of the set of quantum 2-design states is 72 (9 MUB sets each having a cardinality of 8). Determining the real and imaginary parts of $F_{mn}$ respectively for three qubits requires a total of 288 state preparations using the SQPT protocol. Further to estimate the overlap with original state $\Phi _j$, 7 readouts need to be performed. Hence the total number of readouts required to determine a specific element of the three-qubit process matrix with high precision, using the SQPT method, is $288 \times 7 = 2016$. For the MSQPT method, the total number of states we need to prepare are 63 (corresponding to the complete set of basis operators) and the total number of readouts required is 504 (for each MUB set we need to perform 56 readouts, so the total number of readouts is $9 \times 56 = 504$). This makes the MSQPT method vastly more efficient as compared to the standard QPT and SQPT protocols. The experimental complexity and number of ancilla qubits required to determine a specific element of the process matrix with high precision for a three-qubit system, using the MSQPT method, are compared with the standard QPT and SQPT methods in Table 2.

## Conclusions

We proposed a quantum circuit to efficiently implement the MSQPT protocol which reduces the experimental cost of performing standard SQPT. We implemented the MSQPT protocol on the IBM quantum processor. The system was prepared in a mixed state corresponding to all Pauli operators and the MSQPT protocol to perform element wise process tomography of two- and three-qubit quantum gates was successfully implemented. Our experimental results indicate that MSQPT is substantially more efficient as compared to SQPT and standard methods, when estimating specific elements of the process matrix with high precision. We also showed that one can utilize the full process matrix obtained experimentally via MSQPT, to solve the $l_2$-norm minimization problem and reconstruct the underlying true quantum process. The MSQPT method opens up several avenues for future applications such as finding an optimal set of basis operators, developing generalized algorithms to find all sets of quantum maps to perform efficient measurements, and finding efficient decompositions of unitaries using the set of available quantum gates for easy experimental implementation.

## Supplementary Information

Supplementary Information.
