# Supplementary material for: Implementing efficient selective quantum process tomography of superconducting quantum gates on IBM quantum experience
Source: Sci Rep. 2022 Mar 7;12:3688. doi: 10.1038/s41598-022-07721-3 (PMC8901781; doi:10.1038/s41598-022-07721-3)
Supplement: Supplementary file 1 — Supplementary Information. [file 41598_2022_7721_MOESM1_ESM.pdf]

Three-Qubit MSQPT

The list of all five-qubit pure input states $\{|\Omega_i\rangle\}$ is given below.

$$|\Omega_1\rangle = [(0,0,0,\frac{1}{2\sqrt{2}},0,0,0,\frac{1}{2\sqrt{2}},0,0,\frac{1}{2\sqrt{2}},0,0,0,\frac{1}{2\sqrt{2}},0,0,\frac{1}{2\sqrt{2}},0,0,0,\frac{1}{2\sqrt{2}},0,0,\frac{1}{2\sqrt{2}},0,0,0,\frac{1}{2\sqrt{2}},0,0,0)]^T,$$

$$|\Omega_2\rangle = [(0,0,0,-\frac{i}{2\sqrt{2}},0,0,0,\frac{1}{2\sqrt{2}},0,0,-\frac{i}{2\sqrt{2}},0,0,0,\frac{1}{2\sqrt{2}},0,0,-\frac{i}{2\sqrt{2}},0,0,0,\frac{1}{2\sqrt{2}},0,0,-\frac{i}{2\sqrt{2}},0,0,0,\frac{1}{2\sqrt{2}},0,0,0)]^T,$$

$$|\Omega_3\rangle = [(0,0,0,\frac{1}{2},0,0,0,0,0,0,\frac{1}{2},0,0,0,0,0,0,\frac{1}{2},0,0,0,0,0,0,\frac{1}{2},0,0,0,0,0,0,0)]^T,$$

$$|\Omega_4\rangle = [(0,0,0,\frac{1}{2\sqrt{2}},0,0,\frac{1}{2\sqrt{2}},0,0,0,0,\frac{1}{2\sqrt{2}},0,0,\frac{1}{2\sqrt{2}},0,0,\frac{1}{2\sqrt{2}},0,0,\frac{1}{2\sqrt{2}},0,0,0,0,\frac{1}{2\sqrt{2}},0,0,\frac{1}{2\sqrt{2}},0,0,0)]^T,$$

$$|\Omega_5\rangle = [(0,0,\frac{1}{2\sqrt{2}},0,0,0,0,\frac{1}{2\sqrt{2}},0,0,0,\frac{1}{2\sqrt{2}},0,0,\frac{1}{2\sqrt{2}},0,\frac{1}{2\sqrt{2}},0,0,0,0,\frac{1}{2\sqrt{2}},0,0,0,\frac{1}{2\sqrt{2}},0,0,\frac{1}{2\sqrt{2}},0,0,0)]^T,$$

$$|\Omega_6\rangle = [(0,0,-\frac{i}{2\sqrt{2}},0,0,0,0,\frac{i}{2\sqrt{2}},0,0,0,\frac{1}{2\sqrt{2}},0,0,\frac{1}{2\sqrt{2}},0,-\frac{i}{2\sqrt{2}},0,0,0,0,\frac{i}{2\sqrt{2}},0,0,0,\frac{1}{2\sqrt{2}},0,0,\frac{1}{2\sqrt{2}},0,0,0)]^T,$$

$$|\Omega_7\rangle = [(0,0,0,\frac{1}{2\sqrt{2}},0,0,-\frac{1}{2\sqrt{2}},0,0,0,0,\frac{1}{2\sqrt{2}},0,0,\frac{1}{2\sqrt{2}},0,0,\frac{1}{2\sqrt{2}},0,0,-\frac{1}{2\sqrt{2}},0,0,0,0,\frac{1}{2\sqrt{2}},0,0,\frac{1}{2\sqrt{2}},0,0,0)]^T,$$

$$|\Omega_8\rangle = [(0,0,0,-\frac{i}{2\sqrt{2}},0,0,-\frac{i}{2\sqrt{2}},0,0,0,0,\frac{1}{2\sqrt{2}},0,0,\frac{1}{2\sqrt{2}},0,0,-\frac{i}{2\sqrt{2}},0,0,-\frac{i}{2\sqrt{2}},0,0,0,0,\frac{1}{2\sqrt{2}},0,0,\frac{1}{2\sqrt{2}},0,0,0)]^T,$$

$$|\Omega_9\rangle = [(0,0,-\frac{i}{2\sqrt{2}},0,0,0,0,-\frac{i}{2\sqrt{2}},0,0,0,\frac{1}{2\sqrt{2}},0,0,\frac{1}{2\sqrt{2}},0,-\frac{i}{2\sqrt{2}},0,0,0,0,-\frac{i}{2\sqrt{2}},0,0,0,\frac{1}{2\sqrt{2}},0,0,\frac{1}{2\sqrt{2}},0,0,0)]^T,$$

$$|\Omega_{10}\rangle = [(0,0,-\frac{1}{2\sqrt{2}},0,0,0,0,\frac{1}{2\sqrt{2}},0,0,0,\frac{1}{2\sqrt{2}},0,0,\frac{1}{2\sqrt{2}},0,-\frac{1}{2\sqrt{2}},0,0,0,0,\frac{1}{2\sqrt{2}},0,0,0,\frac{1}{2\sqrt{2}},0,0,\frac{1}{2\sqrt{2}},0,0,0)]^T,$$

$$|\Omega_{11}\rangle = [(0,0,0,-\frac{i}{2\sqrt{2}},0,0,\frac{i}{2\sqrt{2}},0,0,0,0,\frac{1}{2\sqrt{2}},0,0,\frac{1}{2\sqrt{2}},0,0,-\frac{i}{2\sqrt{2}},0,0,\frac{i}{2\sqrt{2}},0,0,0,0,\frac{1}{2\sqrt{2}},0,0,\frac{1}{2\sqrt{2}},0,0,0)]^T,$$

$$|\Omega_{12}\rangle = [(0,0,0,\frac{1}{2},0,0,\frac{1}{2},0,0,0,0,0,0,0,0,0,0,\frac{1}{2},0,0,\frac{1}{2},0,0,0,0,0,0,0,0,0,0,0)]^T,$$

$$|\Omega_{13}\rangle = [(0,0,0,\frac{1}{2\sqrt{2}},0,0,0,\frac{1}{2\sqrt{2}},0,0,-\frac{1}{2\sqrt{2}},0,0,0,\frac{1}{2\sqrt{2}},0,0,\frac{1}{2\sqrt{2}},0,0,0,\frac{1}{2\sqrt{2}},0,0,-\frac{1}{2\sqrt{2}},0,0,0,\frac{1}{2\sqrt{2}},0,0,0)]^T,$$

$$|\Omega_{14}\rangle = [(0,0,0,\frac{1}{2\sqrt{2}},0,0,0,\frac{1}{2\sqrt{2}},0,0,-\frac{1}{2\sqrt{2}},0,0,0,\frac{1}{2\sqrt{2}},0,0,\frac{1}{2\sqrt{2}},0,0,0,\frac{1}{2\sqrt{2}},0,0,-\frac{1}{2\sqrt{2}},0,0,0,\frac{1}{2\sqrt{2}},0,0,0)]^T,$$

$$|\Omega_{15}\rangle = [(0,0,0,\frac{1}{2},0,0,0,0,0,0,0,0,0,0,\frac{1}{2},0,0,\frac{1}{2},0,0,0,0,0,0,0,0,0,0,\frac{1}{2},0,0,0)]^T,$$

$$|\Omega_{16}\rangle = [(0,0,0,\frac{1}{2\sqrt{2}},0,0,\frac{1}{2\sqrt{2}},0,0,\frac{1}{2\sqrt{2}},0,0,\frac{1}{2\sqrt{2}},0,0,0,0,0,0,\frac{1}{2\sqrt{2}},0,0,\frac{1}{2\sqrt{2}},0,0,\frac{1}{2\sqrt{2}},0,0,\frac{1}{2\sqrt{2}},0,0,0)]^T,$$

$$|\Omega_{17}\rangle = [(0,0,\frac{1}{2\sqrt{2}},0,0,0,0,\frac{1}{2\sqrt{2}},\frac{1}{2\sqrt{2}},0,0,0,0,\frac{1}{2\sqrt{2}},0,0,0,0,0,0,\frac{1}{2\sqrt{2}},0,0,\frac{1}{2\sqrt{2}},0,0,\frac{1}{2\sqrt{2}},0,0,\frac{1}{2\sqrt{2}},0,0,0)]^T,$$

$$|\Omega_{18}\rangle = [(0,0,-\frac{i}{2\sqrt{2}},0,0,0,0,\frac{i}{2\sqrt{2}},-\frac{i}{2\sqrt{2}},0,0,0,0,\frac{i}{2\sqrt{2}},0,0,0,0,0,0,\frac{1}{2\sqrt{2}},0,0,\frac{1}{2\sqrt{2}},0,0,\frac{1}{2\sqrt{2}},0,0,\frac{1}{2\sqrt{2}},0,0,0)]^T,$$

$$|\Omega_{19}\rangle = [(0,0,0,\frac{1}{2\sqrt{2}},0,0,-\frac{1}{2\sqrt{2}},0,0,\frac{1}{2\sqrt{2}},0,0,-\frac{1}{2\sqrt{2}},0,0,0,0,0,0,\frac{1}{2\sqrt{2}},0,0,\frac{1}{2\sqrt{2}},0,0,\frac{1}{2\sqrt{2}},0,0,\frac{1}{2\sqrt{2}},0,0,0)]^T,$$

$$|\Omega_{20}\rangle = [(0,\frac{1}{2\sqrt{2}},0,0,\frac{1}{2\sqrt{2}},0,0,0,0,0,0,\frac{1}{2\sqrt{2}},0,0,\frac{1}{2\sqrt{2}},0,0,0,0,\frac{1}{2\sqrt{2}},0,0,\frac{1}{2\sqrt{2}},0,0,\frac{1}{2\sqrt{2}},0,0,\frac{1}{2\sqrt{2}},0,0,0)]^T,$$

$$|\Omega_{21}\rangle = [(\frac{1}{2\sqrt{2}},0,0,0,0,\frac{1}{2\sqrt{2}},0,0,0,0,\frac{1}{2\sqrt{2}},0,0,0,0,\frac{1}{2\sqrt{2}},0,0,0,\frac{1}{2\sqrt{2}},0,0,\frac{1}{2\sqrt{2}},0,0,\frac{1}{2\sqrt{2}},0,0,\frac{1}{2\sqrt{2}},0,0,0)]^T,$$

$$|\Omega_{22}\rangle = [(-\frac{i}{2\sqrt{2}},0,0,0,0,\frac{i}{2\sqrt{2}},0,0,0,0,-\frac{i}{2\sqrt{2}},0,0,0,0,\frac{i}{2\sqrt{2}},0,0,0,\frac{1}{2\sqrt{2}},0,0,\frac{1}{2\sqrt{2}},0,0,\frac{1}{2\sqrt{2}},0,0,\frac{1}{2\sqrt{2}},0,0,0)]^T,$$

$$|\Omega_{23}\rangle = [(0,\frac{1}{2\sqrt{2}},0,0,-\frac{1}{2\sqrt{2}},0,0,0,0,0,0,\frac{1}{2\sqrt{2}},0,0,-\frac{1}{2\sqrt{2}},0,0,0,0,\frac{1}{2\sqrt{2}},0,0,\frac{1}{2\sqrt{2}},0,0,\frac{1}{2\sqrt{2}},0,0,\frac{1}{2\sqrt{2}},0,0,0)]^T,$$

$$|\Omega_{24}\rangle = [(0,-\frac{i}{2\sqrt{2}},0,0,-\frac{i}{2\sqrt{2}},0,0,0,0,0,0,\frac{i}{2\sqrt{2}},0,0,\frac{i}{2\sqrt{2}},0,0,0,0,\frac{1}{2\sqrt{2}},0,0,\frac{1}{2\sqrt{2}},0,0,\frac{1}{2\sqrt{2}},0,0,\frac{1}{2\sqrt{2}},0,0,0)]^T,$$

$$|\Omega_{25}\rangle = [(-\frac{i}{2\sqrt{2}},0,0,0,0,-\frac{i}{2\sqrt{2}},0,0,0,0,\frac{i}{2\sqrt{2}},0,0,0,0,\frac{i}{2\sqrt{2}},0,0,0,\frac{1}{2\sqrt{2}},0,0,\frac{1}{2\sqrt{2}},0,0,\frac{1}{2\sqrt{2}},0,0,\frac{1}{2\sqrt{2}},0,0,0)]^T,$$

$$|\Omega_{26}\rangle = [(-\frac{1}{2\sqrt{2}},0,0,0,0,\frac{1}{2\sqrt{2}},0,0,0,0,\frac{1}{2\sqrt{2}},0,0,0,0,-\frac{1}{2\sqrt{2}},0,0,0,\frac{1}{2\sqrt{2}},0,0,\frac{1}{2\sqrt{2}},0,0,\frac{1}{2\sqrt{2}},0,0,\frac{1}{2\sqrt{2}},0,0,0)]^T,$$

$$|\Omega_{27}\rangle = [(0,-\frac{i}{2\sqrt{2}},0,0,\frac{i}{2\sqrt{2}},0,0,0,0,0,0,\frac{i}{2\sqrt{2}},0,0,-\frac{i}{2\sqrt{2}},0,0,0,0,\frac{1}{2\sqrt{2}},0,0,\frac{1}{2\sqrt{2}},0,0,\frac{1}{2\sqrt{2}},0,0,\frac{1}{2\sqrt{2}},0,0,0)]^T,$$

$$|\Omega_{28}\rangle = [(0,0,0,\frac{1}{2\sqrt{2}},0,0,\frac{1}{2\sqrt{2}},0,0,-\frac{1}{2\sqrt{2}},0,0,-\frac{1}{2\sqrt{2}},0,0,0,0,0,0,\frac{1}{2\sqrt{2}},0,0,\frac{1}{2\sqrt{2}},0,0,\frac{1}{2\sqrt{2}},0,0,\frac{1}{2\sqrt{2}},0,0,0)]^T,$$

$$|\Omega_{29}\rangle = [(0,0,\frac{1}{2\sqrt{2}},0,0,0,0,\frac{1}{2\sqrt{2}},-\frac{1}{2\sqrt{2}},0,0,0,0,-\frac{1}{2\sqrt{2}},0,0,0,0,0,\frac{1}{2\sqrt{2}},0,0,\frac{1}{2\sqrt{2}},0,0,\frac{1}{2\sqrt{2}},0,0,\frac{1}{2\sqrt{2}},0,0,0)]^T,$$

$$|\Omega_{30}\rangle = [(0,0,-\frac{i}{2\sqrt{2}},0,0,0,0,\frac{i}{2\sqrt{2}},\frac{i}{2\sqrt{2}},0,0,0,0,-\frac{i}{2\sqrt{2}},0,0,0,0,0,\frac{1}{2\sqrt{2}},0,0,\frac{1}{2\sqrt{2}},0,0,\frac{1}{2\sqrt{2}},0,0,\frac{1}{2\sqrt{2}},0,0,0)]^T,$$

$$|\Omega_{31}\rangle = [(0,0,0,\frac{1}{2\sqrt{2}},0,0,-\frac{1}{2\sqrt{2}},0,0,-\frac{1}{2\sqrt{2}},0,0,\frac{1}{2\sqrt{2}},0,0,0,0,0,0,\frac{1}{2\sqrt{2}},0,0,\frac{1}{2\sqrt{2}},0,0,\frac{1}{2\sqrt{2}},0,0,\frac{1}{2\sqrt{2}},0,0,0)]^T,$$

$$|\Omega_{32}\rangle = [(0,0,0,-\frac{i}{2\sqrt{2}},0,0,-\frac{i}{2\sqrt{2}},0,0,-\frac{i}{2\sqrt{2}},0,0,-\frac{i}{2\sqrt{2}},0,0,0,0,0,0,\frac{1}{2\sqrt{2}},0,0,\frac{1}{2\sqrt{2}},0,0,\frac{1}{2\sqrt{2}},0,0,\frac{1}{2\sqrt{2}},0,0,0)]^T,$$

$$|\Omega_{33}\rangle = [(0,0,-\frac{i}{2\sqrt{2}},0,0,0,0,-\frac{i}{2\sqrt{2}},-\frac{i}{2\sqrt{2}},0,0,0,0,-\frac{i}{2\sqrt{2}},0,0,0,0,0,\frac{1}{2\sqrt{2}},0,0,\frac{1}{2\sqrt{2}},0,0,\frac{1}{2\sqrt{2}},0,0,\frac{1}{2\sqrt{2}},0,0,0)]^T,$$

$$|\Omega_{34}\rangle = [(0,0,-\frac{1}{2\sqrt{2}},0,0,0,0,\frac{1}{2\sqrt{2}},-\frac{1}{2\sqrt{2}},0,0,0,0,\frac{1}{2\sqrt{2}},0,0,0,0,0,\frac{1}{2\sqrt{2}},0,0,\frac{1}{2\sqrt{2}},0,0,\frac{1}{2\sqrt{2}},0,0,\frac{1}{2\sqrt{2}},0,0,0)]^T,$$

$$|\Omega_{35}\rangle = [(0,0,0,-\frac{i}{2\sqrt{2}},0,0,\frac{i}{2\sqrt{2}},0,0,-\frac{i}{2\sqrt{2}},0,0,\frac{i}{2\sqrt{2}},0,0,0,0,0,0,\frac{1}{2\sqrt{2}},0,0,\frac{1}{2\sqrt{2}},0,0,\frac{1}{2\sqrt{2}},0,0,\frac{1}{2\sqrt{2}},0,0,0)]^T,$$

$$|\Omega_{36}\rangle = [(0,-\frac{i}{2\sqrt{2}},0,0,-\frac{i}{2\sqrt{2}},0,0,0,0,0,0,-\frac{i}{2\sqrt{2}},0,0,-\frac{i}{2\sqrt{2}},0,0,0,0,\frac{1}{2\sqrt{2}},0,0,\frac{1}{2\sqrt{2}},0,0,\frac{1}{2\sqrt{2}},0,0,\frac{1}{2\sqrt{2}},0,0,0)]^T,$$

$$|\Omega_{37}\rangle = [(-\frac{i}{2\sqrt{2}},0,0,0,0,-\frac{i}{2\sqrt{2}},0,0,0,0,-\frac{i}{2\sqrt{2}},0,0,0,0,-\frac{i}{2\sqrt{2}},0,0,0,\frac{1}{2\sqrt{2}},0,0,\frac{1}{2\sqrt{2}},0,0,\frac{1}{2\sqrt{2}},0,0,\frac{1}{2\sqrt{2}},0,0,0)]^T,$$

$$|\Omega_{38}\rangle = [(-\frac{1}{2\sqrt{2}},0,0,0,0,\frac{1}{2\sqrt{2}},0,0,0,0,-\frac{1}{2\sqrt{2}},0,0,0,0,\frac{1}{2\sqrt{2}},0,0,0,\frac{1}{2\sqrt{2}},0,0,\frac{1}{2\sqrt{2}},0,0,\frac{1}{2\sqrt{2}},0,0,\frac{1}{2\sqrt{2}},0,0,0)]^T,$$

$$|\Omega_{39}\rangle = [(0,-\frac{i}{2\sqrt{2}},0,0,\frac{i}{2\sqrt{2}},0,0,0,0,0,0,-\frac{i}{2\sqrt{2}},0,0,\frac{i}{2\sqrt{2}},0,0,0,0,\frac{1}{2\sqrt{2}},0,0,\frac{1}{2\sqrt{2}},0,0,\frac{1}{2\sqrt{2}},0,0,\frac{1}{2\sqrt{2}},0,0,0)]^T,$$

$$|\Omega_{40}\rangle = [(0,-\frac{1}{2\sqrt{2}},0,0,-\frac{1}{2\sqrt{2}},0,0,0,0,0,0,\frac{1}{2\sqrt{2}},0,0,\frac{1}{2\sqrt{2}},0,0,0,0,\frac{1}{2\sqrt{2}},0,0,\frac{1}{2\sqrt{2}},0,0,\frac{1}{2\sqrt{2}},0,0,\frac{1}{2\sqrt{2}},0,0,0)]^T,$$

$$|\Omega_{41}\rangle = [(-\frac{1}{2\sqrt{2}},0,0,0,0,-\frac{1}{2\sqrt{2}},0,0,0,0,\frac{1}{2\sqrt{2}},0,0,0,0,\frac{1}{2\sqrt{2}},0,0,0,\frac{1}{2\sqrt{2}},0,0,\frac{1}{2\sqrt{2}},0,0,\frac{1}{2\sqrt{2}},0,0,\frac{1}{2\sqrt{2}},0,0,0)]^T,$$

$$|\Omega_{42}\rangle = [(\frac{i}{2\sqrt{2}},0,0,0,0,-\frac{i}{2\sqrt{2}},0,0,0,0,-\frac{i}{2\sqrt{2}},0,0,0,0,\frac{i}{2\sqrt{2}},0,0,0,\frac{1}{2\sqrt{2}},0,0,\frac{1}{2\sqrt{2}},0,0,\frac{1}{2\sqrt{2}},0,0,\frac{1}{2\sqrt{2}},0,0,0)]^T,$$

$$|\Omega_{43}\rangle = [(0,-\frac{1}{2\sqrt{2}},0,0,\frac{1}{2\sqrt{2}},0,0,0,0,0,0,\frac{1}{2\sqrt{2}},0,0,-\frac{1}{2\sqrt{2}},0,0,0,0,\frac{1}{2\sqrt{2}},0,0,\frac{1}{2\sqrt{2}},0,0,\frac{1}{2\sqrt{2}},0,0,\frac{1}{2\sqrt{2}},0,0,0)]^T,$$

$$|\Omega_{44}\rangle = [(0,0,0,-\frac{i}{2\sqrt{2}},0,0,-\frac{i}{2\sqrt{2}},0,0,\frac{i}{2\sqrt{2}},0,0,\frac{i}{2\sqrt{2}},0,0,0,0,0,0,\frac{1}{2\sqrt{2}},0,0,\frac{1}{2\sqrt{2}},0,0,\frac{1}{2\sqrt{2}},0,0,\frac{1}{2\sqrt{2}},0,0,0)]^T,$$

$$|\Omega_{45}\rangle = [(0,0,-\frac{i}{2\sqrt{2}},0,0,0,0,-\frac{i}{2\sqrt{2}},\frac{i}{2\sqrt{2}},0,0,0,0,\frac{i}{2\sqrt{2}},0,0,0,0,0,\frac{1}{2\sqrt{2}},0,0,\frac{1}{2\sqrt{2}},0,0,\frac{1}{2\sqrt{2}},0,0,\frac{1}{2\sqrt{2}},0,0,0)]^T,$$

$$|\Omega_{46}\rangle = [(0,0,-\frac{1}{2\sqrt{2}},0,0,0,0,\frac{1}{2\sqrt{2}},\frac{1}{2\sqrt{2}},0,0,0,0,-\frac{1}{2\sqrt{2}},0,0,0,0,0,\frac{1}{2\sqrt{2}},0,0,\frac{1}{2\sqrt{2}},0,0,\frac{1}{2\sqrt{2}},0,0,\frac{1}{2\sqrt{2}},0,0,0)]^T,$$

$$|\Omega_{47}\rangle = [(0,0,0,-\frac{i}{2\sqrt{2}},0,0,\frac{i}{2\sqrt{2}},0,0,\frac{i}{2\sqrt{2}},0,0,-\frac{i}{2\sqrt{2}},0,0,0,0,0,0,\frac{1}{2\sqrt{2}},0,0,\frac{1}{2\sqrt{2}},0,0,\frac{1}{2\sqrt{2}},0,0,\frac{1}{2\sqrt{2}},0,0,0)]^T,$$

$$|\Omega_{48}\rangle = [(0,0,0,\frac{1}{2},0,0,\frac{1}{2},0,0,\frac{1}{2},0,0,\frac{1}{2},0,0,0,0,0,0,0,0,0,0,0,0,0,0,0,0,0,0,0)]^T,$$

$$|\Omega_{49}\rangle = [(0,0,0,\frac{1}{2\sqrt{2}},0,0,0,\frac{1}{2\sqrt{2}},0,0,\frac{1}{2\sqrt{2}},0,0,0,\frac{1}{2\sqrt{2}},0,0,-\frac{1}{2\sqrt{2}},0,0,0,\frac{1}{2\sqrt{2}},0,0,-\frac{1}{2\sqrt{2}},0,0,0,\frac{1}{2\sqrt{2}},0,0,0)]^T,$$

$$|\Omega_{50}\rangle = [(0,0,0,-\frac{i}{2\sqrt{2}},0,0,0,\frac{1}{2\sqrt{2}},0,0,-\frac{i}{2\sqrt{2}},0,0,0,\frac{1}{2\sqrt{2}},0,0,\frac{i}{2\sqrt{2}},0,0,0,\frac{1}{2\sqrt{2}},0,0,\frac{i}{2\sqrt{2}},0,0,0,\frac{1}{2\sqrt{2}},0,0,0)]^T,$$

$$|\Omega_{51}\rangle = [(0,0,0,\frac{1}{2},0,0,0,0,0,0,\frac{1}{2},0,0,0,0,0,0,0,0,0,0,\frac{1}{2},0,0,0,0,0,0,\frac{1}{2},0,0,0)]^T,$$

$$|\Omega_{52}\rangle = [(0,0,0,\frac{1}{2\sqrt{2}},0,0,\frac{1}{2\sqrt{2}},0,0,0,0,\frac{1}{2\sqrt{2}},0,0,\frac{1}{2\sqrt{2}},0,0,-\frac{1}{2\sqrt{2}},0,0,-\frac{1}{2\sqrt{2}},0,0,0,0,\frac{1}{2\sqrt{2}},0,0,\frac{1}{2\sqrt{2}},0,0,0)]^T,$$

$$|\Omega_{53}\rangle = [(0,0,\frac{1}{2\sqrt{2}},0,0,0,0,\frac{1}{2\sqrt{2}},0,0,0,\frac{1}{2\sqrt{2}},0,0,\frac{1}{2\sqrt{2}},0,-\frac{1}{2\sqrt{2}},0,0,0,0,-\frac{1}{2\sqrt{2}},0,0,0,\frac{1}{2\sqrt{2}},0,0,\frac{1}{2\sqrt{2}},0,0,0)]^T,$$

$$|\Omega_{54}\rangle = [(0,0,-\frac{i}{2\sqrt{2}},0,0,0,0,\frac{i}{2\sqrt{2}},0,0,0,\frac{1}{2\sqrt{2}},0,0,\frac{1}{2\sqrt{2}},0,\frac{i}{2\sqrt{2}},0,0,0,0,-\frac{i}{2\sqrt{2}},0,0,0,\frac{1}{2\sqrt{2}},0,0,\frac{1}{2\sqrt{2}},0,0,0)]^T,$$

$$|\Omega_{55}\rangle = [(0,0,0,\frac{1}{2\sqrt{2}},0,0,-\frac{1}{2\sqrt{2}},0,0,0,0,\frac{1}{2\sqrt{2}},0,0,\frac{1}{2\sqrt{2}},0,0,-\frac{1}{2\sqrt{2}},0,0,\frac{1}{2\sqrt{2}},0,0,0,0,\frac{1}{2\sqrt{2}},0,0,\frac{1}{2\sqrt{2}},0,0,0)]^T,$$

$$|\Omega_{56}\rangle = [(0,0,0,-\frac{i}{2\sqrt{2}},0,0,-\frac{i}{2\sqrt{2}},0,0,0,0,\frac{1}{2\sqrt{2}},0,0,\frac{1}{2\sqrt{2}},0,0,\frac{i}{2\sqrt{2}},0,0,\frac{i}{2\sqrt{2}},0,0,0,0,\frac{1}{2\sqrt{2}},0,0,\frac{1}{2\sqrt{2}},0,0,0)]^T,$$

$$|\Omega_{57}\rangle = [(0,0,-\frac{i}{2\sqrt{2}},0,0,0,0,-\frac{i}{2\sqrt{2}},0,0,0,\frac{1}{2\sqrt{2}},0,0,\frac{1}{2\sqrt{2}},0,\frac{i}{2\sqrt{2}},0,0,0,0,\frac{i}{2\sqrt{2}},0,0,0,\frac{1}{2\sqrt{2}},0,0,\frac{1}{2\sqrt{2}},0,0,0)]^T,$$

$$|\Omega_{58}\rangle = [(0,0,-\frac{1}{2\sqrt{2}},0,0,0,0,\frac{1}{2\sqrt{2}},0,0,0,\frac{1}{2\sqrt{2}},0,0,\frac{1}{2\sqrt{2}},0,\frac{1}{2\sqrt{2}},0,0,0,0,-\frac{1}{2\sqrt{2}},0,0,0,\frac{1}{2\sqrt{2}},0,0,\frac{1}{2\sqrt{2}},0,0,0)]^T,$$

$$|\Omega_{59}\rangle = [(0,0,0,-\frac{i}{2\sqrt{2}},0,0,\frac{i}{2\sqrt{2}},0,0,0,0,\frac{1}{2\sqrt{2}},0,0,\frac{1}{2\sqrt{2}},0,0,\frac{i}{2\sqrt{2}},0,0,-\frac{i}{2\sqrt{2}},0,0,0,0,\frac{1}{2\sqrt{2}},0,0,\frac{1}{2\sqrt{2}},0,0,0)]^T,$$

$$|\Omega_{60}\rangle = [(0,0,0,\frac{1}{2},0,0,\frac{1}{2},0,0,0,0,0,0,0,0,0,0,0,0,0,0,0,0,0,0,\frac{1}{2},0,0,\frac{1}{2},0,0,0)]^T,$$

$$|\Omega_{61}\rangle = [(0,0,0,\frac{1}{2\sqrt{2}},0,0,0,\frac{1}{2\sqrt{2}},0,0,-\frac{1}{2\sqrt{2}},0,0,0,\frac{1}{2\sqrt{2}},0,0,-\frac{1}{2\sqrt{2}},0,0,0,\frac{1}{2\sqrt{2}},0,0,\frac{1}{2\sqrt{2}},0,0,0,\frac{1}{2\sqrt{2}},0,0,0)]^T,$$

$$|\Omega_{52}\rangle = [(0,0,0,-\frac{i}{2\sqrt{2}},0,0,0,\frac{1}{2\sqrt{2}},0,0,\frac{i}{2\sqrt{2}},0,0,0,\frac{1}{2\sqrt{2}},0,0,\frac{i}{2\sqrt{2}},0,0,0,\frac{1}{2\sqrt{2}},0,0,-\frac{i}{2\sqrt{2}},0,0,0,\frac{1}{2\sqrt{2}},0,0,0)]^T,$$

$$|\Omega_{63}\rangle = [(0,0,0,\frac{1}{2},0,0,0,0,0,0,0,0,0,0,\frac{1}{2},0,0,0,0,0,0,\frac{1}{2},0,0,\frac{1}{2},0,0,0,0,0,0,0)]^T,$$
